# Cancer Diagnostic and Predictive Biomarkers 2015

**DOI:** 10.1155/2015/235985

**Published:** 2015-10-12

**Authors:** Franco M. Buonaguro, C. David Pauza, Maria Lina Tornesello, Pierre Hainaut, Renato Franco

**Affiliations:** ^1^Unit of Molecular Biology and Viral Oncology, Department of Experimental Oncology, Istituto Nazionale Tumori IRCCS Fondazione Pascale, 80131 Napoli, Italy; ^2^Department of Microbiology and Immunology, Institute of Human Virology, Baltimore, MD 21201, USA; ^3^Department of Research, International Prevention Research Institute, 69006 Lyon, France; ^4^Department of Pathology, Istituto Nazionale Tumori IRCCS Fondazione Pascale, 80131 Napoli, Italy

Early cancer diagnosis and prediction of responsivity to treatments are crucial elements for favorable prognosis and increased effectiveness of therapies in cancer patients. This special issue compiles relevant articles focused on the development of innovative cancer biomarkers and their validation.

Availability of advanced molecular biology tools, including deep-sequencing and RNA-seq, allowed the identification of new unique biomarkers for more sensitive and specific diagnosis and the possibility to select new candidate therapeutic targets for personalized medicine.

The negative prognostic role of stem cell markers, with higher progression rate, has been reported by G. Chene et al. in preinvasive tubal lesions of ovarian carcinoma, F. Collina et al. in triple negative breast cancers, and S. H. Kim et al. in prostate cancer patients, where high level of GAPDH mRNA is a significant predictor of recurrence. The negative prognostic value, particularly for head and neck cancers, of cell-to-cell internalization was reported by M. Schwegler et al., and inverse correlation with expression levels of iNOS was reported by L. Yang et al.

Different biomarkers have been analyzed for early diagnosis and follow-up monitoring spanning within serum detection of glycosylated biomarkers (described by A. Kirwan et al.), serum levels of 15-F2t-isoprostane in non-melanoma skin cancer (B. Freitas et al.), and methylation levels of GALR1 promoter in endometrial lesions by pyrosequencing analysis (C. Kottaridi et al.). Early diagnosis of liver metastasis by CT perfusion has been reported by Y. Wang et al.

Predictive therapeutic biomarkers have been described to chemoradiation in locally advanced rectal cancer by R. Conde-Muíño et al. and to radiotherapy in localized prostate cancer by A. Wilkins et al.

The role of miRNAs in cancers and their prognostics relevance has been analyzed by A. Min et al. in oral carcinogenesis. J. Long et al. have focused on miRNA 193b in human breast cancer, and S. Wu et al. on miRNA 125b in gastric cancer.

Finally identification of HHV-8 sequences in conjunctival neoplasia of Uganda patients offers the possibility to unveil a further cancer association to such herpesvirus and highlights the need to look for indirect role of virus with low-modest oncogenic potential in immunodeficient patients (including low risk HPVs in HIV-positive patients).

By compiling these papers, we hope to enrich our readers and researchers with respect to the current wide range of cancer biomarkers, actively pursued by several worldwide research groups.

*Franco M. Buonaguro* *C. David Pauza* *Maria Lina Tornesello* *Pierre Hainaut* *Renato Franco*

